# Characteristics of specialists treating hypothyroid patients: the “THESIS” collaborative

**DOI:** 10.3389/fendo.2023.1225202

**Published:** 2023-11-07

**Authors:** Miloš Žarković, Roberto Attanasio, Endre V. Nagy, Roberto Negro, Enrico Papini, Petros Perros, Chagit Adler Cohen, Ersin Akarsu, Maria Alevizaki, Göksun Ayvaz, Tomasz Bednarczuk, Eszter Berta, Miklos Bodor, Anna Maria Borissova, Mihail Boyanov, Camille Buffet, Maria-Cristina Burlacu, Jasmina Ćirić, Juan J. Díez, Harald Dobnig, Valentin Fadeyev, Benjamin C. T. Field, Eric Fliers, Jacob Stampe Frølich, Dagmar Führer, Juan Carlos Galofré, Tommi Hakala, Jan Jiskra, Peter Kopp, Michael Krebs, Michal Kršek, Martin Kužma, Mikael Lantz, Ivica Lazúrová, Laurence Leenhardt, Vitaliy Luchytskiy, Anne McGowan, Miguel Melo, Saara Metso, Carla Moran, Tatyana Morgunova, Tronko Mykola, Biljana Nedeljković Beleslin, Dan Alexandru Niculescu, Božidar Perić, Tereza Planck, Catalina Poiana, Francisca Marques Puga, Eyal Robenshtok, Patrick Rosselet, Marek Ruchala, Kamilla Ryom Riis, Alla Shepelkevich, David Unuane, Irfan Vardarli, W. Edward Visser, Andromachi Vrionidou, Younes R. Younes, Elena Yurenya, Laszlo Hegedüs

**Affiliations:** ^1^ University of Belgrade Faculty of Medicine, Internal Medicine, Belgrade, Serbia; ^2^ Clinic of Endocrinology, Diabetes and Diseases of Metabolism, Thyroid Department, Belgrade, Serbia; ^3^ Associazione Medici Endocrinologi, Scientific Committee, Milan, Italy; ^4^ Division of Endocrinology, Department of Medicine, Faculty of Medicine, University of Debrecen, Debrecen, Hungary; ^5^ Ospedale Vito Fazzi, Department of Endocrinology, Lecce, Italy; ^6^ Department of Endocrinology and Metabolism, Regina Apostolorum Hospital, Albano Laziale, Roma, Italy; ^7^ Institute of Translational and Clinical Research, Newcastle University, Newcastle Upon Tyne, United Kingdom; ^8^ Rabin Medical Center, Tel Aviv University, Tel Aviv, Israel; ^9^ Department of Internal Medicine, Division of Endocrinology, Faculty of Medicine, Gaziantep University, Gaziantep, Türkiye; ^10^ Endocrine Unit and Diabetes Centre, Department of Clinical Therapeutics, Alexandra Hospital, National and Kapodistrian University of Athens Faculty of Medicine, Athens, Greece; ^11^ Department of Endocrinology and Metabolism, Koru Ankara Hospital, Ankara, Türkiye; ^12^ Department of Internal Medicine and Endocrinology, Medical University of Warsaw, Warsaw, Poland; ^13^ Sofia University Saint Kliment Ohridski, Medical Faculty, Clinic of Endocrinology and Metabolism, University Hospital “Sofiamed”, Sofia, Bulgaria; ^14^ University Hospital Alexandrovska, Clinic of Endocrinology and Metabolism, Medical University-Sofia, Internal Medicine, Sofia, Bulgaria; ^15^ GRC n 16, GRC Thyroid Tumors, Thyroid Disease and Endocrine Tumor Department, AP-HP, Hôpital Pitié Salpêtrière, Sorbonne University, Paris, France; ^16^ Department of Endocrinology and Nutrition, Cliniques Universitaires St-Luc, Université Catholique De Louvain, Brussels, Belgium; ^17^ Department of Endocrinology, Hospital Universitario Puerta De Hierro Majadahonda, Madrid, Spain; ^18^ Instituto De Investigación Sanitaria Puerta De Hierro Segovia De Arana, Majadahonda, Madrid, Spain; ^19^ Department of Medicine, Universidad Autónoma De Madrid, Madrid, Spain; ^20^ Thyroid Endocrinology, Osteoporosis Institute Dobnig, Graz, Austria; ^21^ Department of Endocrinology No. 1, N.V. Sklifosovsky Institute of Clinical Medicine, I.M. Sechenov First Moscow State Medical University, Moscow, Russia; ^22^ University of Surrey Faculty of Health and Medical Sciences, Section of Clinical Medicine, Prague, United Kingdom; ^23^ Department of Endocrinology & Metabolism, Amsterdam UMC, University of Amsterdam, Amsterdam, Netherlands; ^24^ Department of Endocrinology, Odense University Hospital, Odense, Denmark; ^25^ University Hospital Essen, Department of Endocrinology, Diabetes and Metabolism, University-Duisburg-Essen, Essen, Germany; ^26^ Departmento De Endocrinologia e Nutrición, Clínica Universidad De Navarra, Pamplona, Spain; ^27^ Department of Surgery, Tampere University Hospital, Tampere, Finland; ^28^ 3rd Department of Medicine, 1st Faculty of Medicine, Charles University, General University Hospital, Prague, Czechia; ^29^ Division of Endocrinology, Diabetology and Metabolism, University of Lausanne, Lausanne, Switzerland; ^30^ Department of Medicine III, Division of Endocrinology, Medical University of Vienna, Vienna, Austria; ^31^ 5th Department of Internal Medicine, Medical Faculty of Commenius University and University Hospital, Bratislava, Slovakia; ^32^ Department of Endocrinology, Skåne University Hospital, Malmö, Sweden; ^33^ P. J. Šafárik University Košice, 1st Department of Internal Medicine of the Medical Faculty, Košice, Slovakia; ^34^ Hopital Pitie-Salpetriere, Thyroid and Endocrine Tumors Unit, Institut of Endocrinology, Sorbonne University, Paris, France; ^35^ Department of Reproductive Endocrinogy, Institute of Endocrinology and Metabolism named after V.P. Komissarenko, National Academy of Medical Science of Ukraine, Kyiv, Ukraine; ^36^ Robert Graves Institute, Tallaght University Hospital, Dublin, Ireland; ^37^ Department of Endocrinology, Diabetes and Metabolism, Medical Faculty, University of Coimbra, Coimbra, Portugal; ^38^ Department of Internal Medicine, Tampere University Hospital, Tampere, Finland; ^39^ Diabetes & Endocrinology Section, Beacon Hospital, Dublin, Ireland; ^40^ School of Medicine, University College Dublin, Dublin, Ireland; ^41^ Institute of Endocrinology and Metabolism named after V.P. Komissarenko, National Academy of Medical Science of Ukraine, Kyiv, Ukraine; ^42^ Department of Endocrinology, Carol Davila University of Medicine and Pharmacy, Bucharest, Romania; ^43^ Department of Endocrinology, Diabetes and Metabolic Diseases “Mladen Sekso”, University Hospital Center “Sisters of Mercy”, Zagreb, Croatia; ^44^ Endocrinology, Diabetes and Metabolism Service, Porto Hospital and University Centre, Porto, Portugal; ^45^ Endocrinology Institute, Rabin Medical Center, Tel Aviv University Sackler Faculty of Medicine, Tel Aviv, Israel; ^46^ Cabinet Médical, 2, Rue Bellefontaine, Lausanne, Switzerland; ^47^ Department of Endocrinology, Metabolism and Internal Medicine, Poznan University of Medical Sciences, Poznan, Poland; ^48^ Department of Endocrinology, Belarusian State Medical University, Minsk, Belarus; ^49^ Department of Internal Medicine, Endocrine Unit, UZ Brussel, Vrije Universiteit Brussel, Brussel, Belgium; ^50^ Department of Medicine I, Klinikum Vest GmbH, Knappschaftskrankenhaus Recklinghausen, Recklinghausen, Germany; ^51^ 5th Medical Department, Division of Endocrinology and Diabetes, Medical Faculty Mannheim, Heidelberg University, Mannheim, Germany; ^52^ Rotterdam Thyroid Center, Department of Internal Medicine, Erasmus MC, Rotterdam, Netherlands; ^53^ Department of Endocrinology and Diabetes Centre, Hellenic Red Cross Hospital, Athens, Greece; ^54^ East Surrey Hospital, Surrey & Sussex Healthcare NHS Trust, Redhill, Surrey, United Kingdom; ^55^ Minsk Endocrinology Medical Center, Minsk, Belarus

**Keywords:** hypothyroidism, questionnaire, endocrinologists, healthcare delivery, Europe

## Abstract

**Introduction:**

Thyroid specialists influence how hypothyroid patients are treated, including patients managed in primary care. Given that physician characteristics influence patient care, this study aimed to explore thyroid specialist profiles and associations with geo-economic factors.

**Methods:**

Thyroid specialists from 28 countries were invited to respond to a questionnaire, Treatment of Hypothyroidism in Europe by Specialists: an International Survey (THESIS). Geographic regions were defined according to the United Nations Statistics Division. The national economic status was estimated using World Bank data on the gross national income per capita (GNI per capita).

**Results:**

5,695 valid responses were received (response rate 33·0%). The mean age was 49 years, and 65·0% were female. The proportion of female respondents was lowest in Northern (45·6%) and highest in Eastern Europe (77·2%) (p <0·001). Respondent work volume, university affiliation and private practice differed significantly between countries (p<0·001). Age and GNI per capita were correlated inversely with the proportion of female respondents (p<0·01). GNI per capita was inversely related to the proportion of respondents working exclusively in private practice (p<0·011) and the proportion of respondents who treated >100 patients annually (p<0·01).

**Discussion:**

THESIS has demonstrated differences in characteristics of thyroid specialists at national and regional levels, strongly associated with GNI per capita. Hypothyroid patients in middle-income countries are more likely to encounter female thyroid specialists working in private practice, with a high workload, compared to high-income countries. Whether these differences influence the quality of care and patient satisfaction is unknown, but merits further study.

## 1 Introduction

Hypothyroidism is a common condition, with a prevalence of overt and subclinical disease of 0·2 and 5·3%, respectively, and its prevalence is rising (1). Unfortunately, in real life, almost half of the patients do not achieve therapeutic targets (2). A serum TSH outside the normal range while on thyroxine (L-T4) replacement is associated with increased morbidity and mortality (3). Therefore, a significant burden of disease is associated with hypothyroidism, which can be addressed with very cheap interventions (adjustment of L-T4 dose and additional monitoring). However, implementation is vitally dependent on appropriate medical supervision. Failure to achieve a normal serum TSH is a common reason for hypothyroid patients on L-T4 being referred to thyroid specialists (4, 5).

The available information on the characteristics of specialists who treat patients with hypothyroidism in Europe is scanty. A survey of members of the European Society of Endocrinology (ESE) revealed that thyroid disorders comprised 28% of the endocrinologist workload (6). Details regarding the characteristics of specialists treating thyroid diseases were unavailable, and have not emerged since. Furthermore, it is notable that in addition to endocrinologists, clinicians from several other specialities not represented in the ESE survey treat patients with hypothyroidism in Europe. Regional differences in managing some thyroid conditions have been reported (7). However, hypothyroidism has not been studied in this context except for one previous survey, which found that American physicians were more likely to prescribe L-T3 and desiccated thyroid extract (DTE) than their European counterparts (8). The role of thyroid specialists in managing this wave of demands on healthcare systems is likely to be important. Also, primary care physicians tend to follow the lead of specialists (9). Europe comprises countries with differences in healthcare provision, patient demographics, epidemiology of thyroid disease, and gross national income per capita (GNI per capita); therefore, further exploration of these variables can help understand, plan, and improve healthcare delivery (10).

THESIS (“Treatment of Hypothyroidism in Europe by Specialists: An International Survey”) was a survey of European specialists treating patients with hypothyroidism. One of the study aims was to document the demographic and work-related characteristics of specialists who treat hypothyroid patients in European countries. Twenty out of twenty eight countries surveyed have already reported their national data (11–30). Differences in healthcare delivery may impact the patient experience, and therefore comparisons between regions and countries may provide valuable insights. Here, we present the aggregate data from THESIS and explore demographic, work-related and geo-economic characteristics of specialists treating hypothyroidism in Europe.

## 2 Materials and methods

The THESIS survey was conducted according to the checklist for reporting results of Internet-based e-surveys (CHERRIES) (31). The target population consisted of members of national endocrine and/or thyroid related professional organisations who treated patients with hypothyroidism and comprised primarily endocrinologists, nuclear medicine physicians, and internists. This was a convenience sample (participants were selected through “convenient” data sources for researchers). Project oversight was provided by a Steering Committee (LH, EVN, EP, PP, RA and RN).

### 2.1 Questionnaire

The THESIS questionnaire was developed in English. It was initially tested in a pilot study of Italian endocrinologists after translation into Italian, following which it underwent revisions to reach its final form (22). The questionnaire included eight questions about the responding physician and twenty-three questions concerning the use of thyroid hormones in different clinical settings (see Appendix). Completion of the survey required less than 15 minutes. National leads had a choice of using the original English version or translations. Translations were performed by a designated bilingual clinician and verified by another bilingual senior clinician. Twelve countries adopted the original English version (Belgium, Denmark, Finland, Greece, Ireland, Israel, Netherlands, Romania, Slovakia, Sweden, Switzerland and the United Kingdom). Fifteen countries translated it into their local language (Austria, Belarus, Bulgaria, Croatia, Czech Republic, France, Germany, Hungary, Italy, Poland, Portugal, Russia, Serbia, Turkey and Ukraine). The Spanish survey was offered to members of the Spanish Society of Endocrinology in both the original English and its Spanish translation. The national leads decided how to distribute the questionnaire to thyroid specialists in their country. Online platforms were used in twenty-six countries, namely Lime Survey in twelve (Croatia, Denmark, Finland, Greece, Hungary, Ireland, Italy, Poland, Romania, Russia, Serbia and Ukraine), SurveyMonkey in nine (Austria, Belgium, Czech Republic, France, Germany, Netherlands, Slovakia, Sweden and Switzerland), Google Forms in four (Israel, Portugal, Spain, Turkey) and Qualtrics by one (United Kingdom), while e-mails were used in Belarus and Bulgaria.

### 2.2 Selection criteria

European countries fulfilling the following criteria were targeted: a population of at least 4 million, having a national endocrine and/or thyroid professional organisation and a national medical journal. The study aimed for a minimum of 100 respondents from each country. Eligible national endocrine and thyroid professional organisations were approached by the Steering Committee and were invited to participate. Two endocrinologists per country were identified as national leads. All members of the national endocrine and thyroid professional organisations were invited to participate by an e-mail from the President of each organisation, including an electronic link to the questionnaire.

### 2.3 Survey and data management

Responses on the online platforms were collected anonymously or anonymised when received by e-mail. The THESIS survey started in March 2019 and ended in April 2021. The survey was kept open for a median of 7 weeks (range 3-20) in each country. Respondents agreed to fill out the survey voluntarily, were aware that they could at any point leave the survey and did not receive any incentives. Personally identifiable data were not collected. The national leads and Steering Committee were responsible for data integrity and safekeeping for locally collected and aggregated data, respectively. An institutional board review was not necessary as the survey was anonymous.

### 2.4 Statistical analyses

Only data from respondents who completed all questions about demographic data were considered valid for statistical analyses. For statistical analyses, R was used (32). Survey data were not weighted. Qualitative variables were reported using frequencies and proportions, and quantitative variables using means and standard deviations. Chi-square and Cramer’s tests were used to test the association between qualitative variables. Statistical analyses were also performed using linear, logistic, and ordinal regression as appropriate (33), using R packages statistics and ordinal. The level of statistical significance was fixed at 5%. The effect size is independent of sample size and p-value and allows disregarding statistically significant but practically irrelevant results (34, 35). Therefore, we report both the p-value and effect size measures and are guided by the effect size measures. Cramer’s V measures effect size, and values were interpreted according to Rea and Parker (34). Cramer’s V values less than 0·1 are interpreted as negligible, between 0·1 and 0·2 as weak, 0·2-0·4 as moderate, 0·4-0·6 as relatively strong, and over 0·6 as a strong association. When necessary, interval variables, such as age, that were aggregated into the groups were converted to means using the R package actuar (36).

Geographic regions were defined according to the United Nations Statistics Division definition (37) (Eastern Europe: Belarus, Bulgaria, Czech Republic, Hungary, Poland, Romania, Russian Federation, Slovakia, Ukraine; Northern Europe: Denmark, Finland, Ireland, Sweden, United Kingdom; Southern Europe: Croatia, Greece, Italy, Portugal, Serbia, Slovenia, Spain; Western Europe: Austria, Belgium, France, Germany, Netherlands, Switzerland; Western Asia: Israel, Turkey). Data on GNI per capita in US dollars were derived from the World Bank (2019, Atlas Method) (38).

## 3 Results

### 3.1 Responses

**Figure 1:**
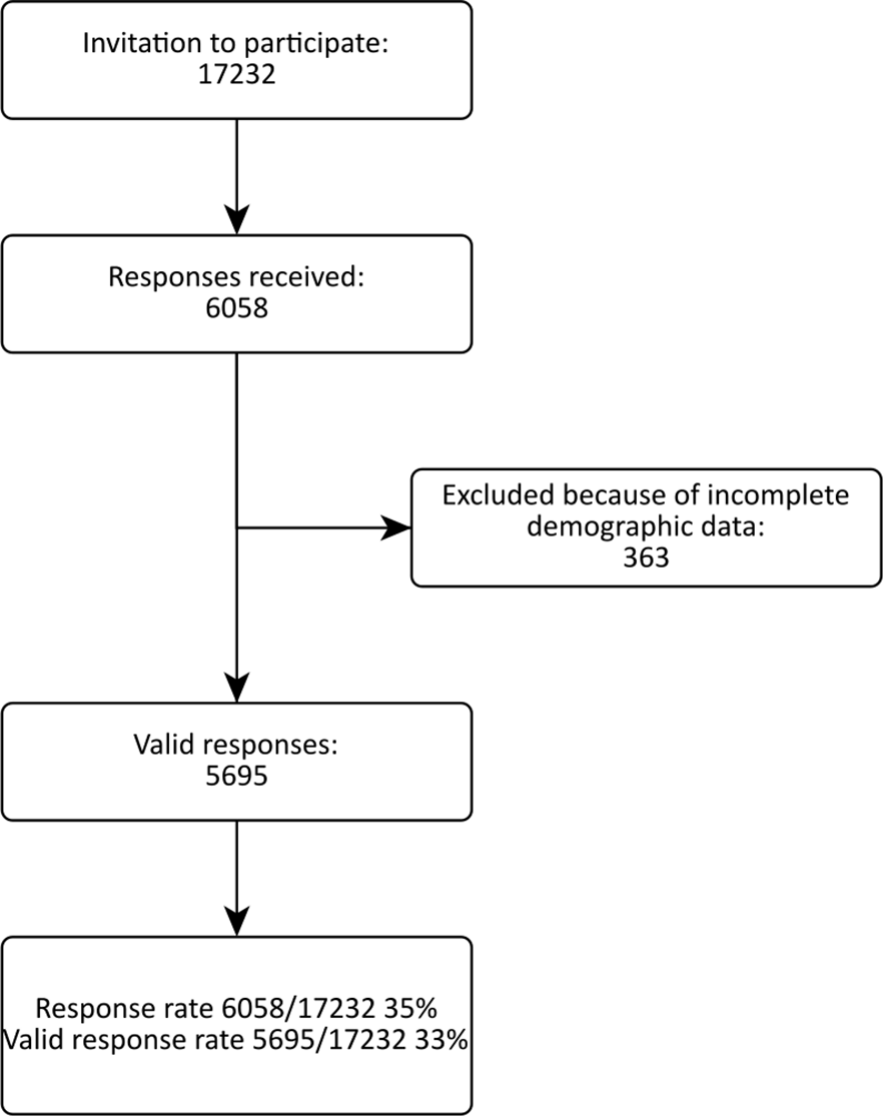
Flow chart of the THESIS survey.

**Figure 2:**
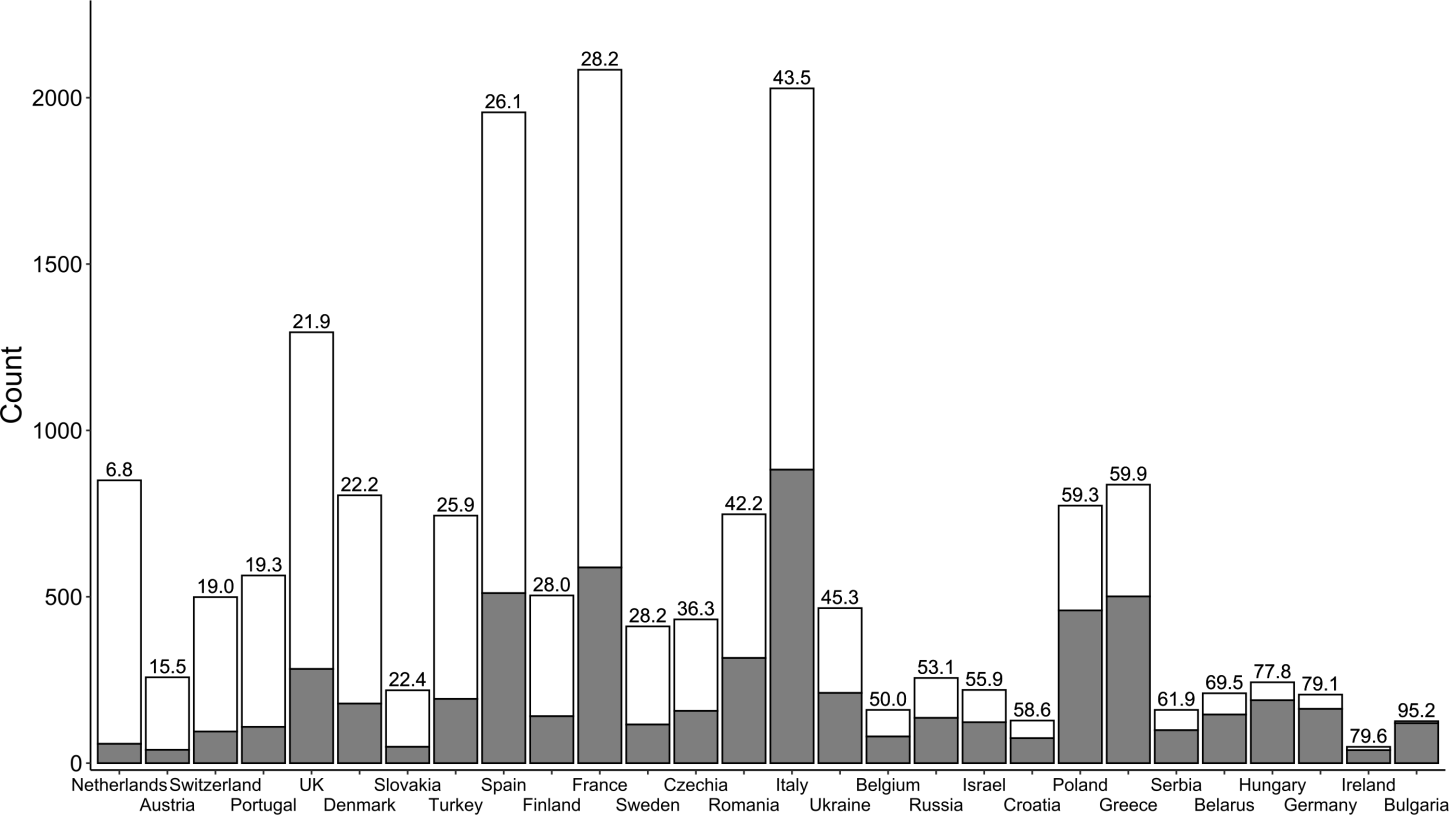
Responses to the questionnaire by country. The height of the bars represents the total number of invitees. The number of respondents is grey, and the response rate (%) is the number on the top of each bar. Bars are sorted by the response rate, from low to high.

A total of 17,232 invitations led to 6,058 responses, of which 5,695 were valid (**Figure 1**). The overall valid response rate was 33·0%. The median response rate per country was 40·6% (range 6·8-95·2%, first quartile 25·0%, third quartile 51·4%). Twenty-eight of the 29 invited countries (all in Europe plus Turkey and Israel) agreed to participate, with Norway being the only nation that declined. Response rates significantly differed between countries, ranging from 6·8-95·2% (p <0·01, **Figure 2**).

### 3.2 Baseline characteristics

The baseline characteristics of respondents are shown in **Table 1**. The mean age was 49.1 (SD 12.0) years, and 65% were female. Most respondents (98.0%) were working at the patients and 2% were basic scientists, and 93.0% were endocrinologists. Only 9·6% were members of international professional thyroid organisations. A substantial subgroup (38·3%) practised at a university centre, and 16·2% exclusively in private practice. Nearly two-thirds (62·1%) of respondents treated more than 100 hypothyroid patients per year.

**Table 1:** Baseline characteristics of respondents.

Variable	Number (%)
Clinical or Research	
Clinical	5,583 (98·0)
Research	112 (2·0)
Total	5,695
Sex	
Female	3,700 (65·0)
Male	1,995 (35·0)
Total	5,695
Age (years)	
≤30	282 (5·0)
31-40	1,375 (24·1)
41-50	1,565 (27·5)
51-60	1,479 (26·0)
61-70	792 (13·9)
70+	202 (3·5)
Total	5,695
Endocrinologist	
Yes	5,299 (93·0)
No	396 (7·0)
Total	5,695
ETA/ATA/LATS/AOTA Member^1^	
Yes	544 (9·6)
No	5,151 (90·4)
Total	5,695
Practice at University Centre	
Yes	2,181 (38·3)
No	3,514 (61·7)
Total	5,695
Exclusively in private practice	
Yes	794 (16·2)
No	4,097 (83·8)
Total^2^	4,891
Hypothyroid patients per year	
Fewer than 10	158 (2·8)
10-50	787 (13·9)
51-100	1,206 (21·2)
More than 100	3,526 (62·1)
Total^3^	5,677

^1^ETA, European Thyroid Association; ATA, American Thyroid Association; LATS, Latin American Thyroid Society; AOTA, Asia and Oceania Thyroid Association. ^2^Respondents working both in private practice and in the public healthcare sector were excluded from the analysis. ^3^Some respondents did not answer this question.

#### 3.2.1 Sex

The sex distribution of respondents in our sample was comparable to the sex distribution of the European physicians. Data on the sex distribution of European physicians were obtained from Eurostat, the statistical office of the European Union (39) (largest Cramer’s v 0·027). Sixty-five per cent of THESIS respondents were female, ranging between 36·6-93·9% among different countries. The association between sex distribution and the country was moderate (p < 0·001, Cramer’s v 0·28). The proportion of female respondents differed significantly between regions with a moderate association (p < 0·001, Cramer’s v 0·21) and was lowest in Northern Europe (45·6%) and highest in Eastern Europe (77·2%). Younger age correlated significantly with an increased proportion of female respondents (Cochran-Armitage test for trend, p-value <0·001, two-sided). To explore the impact of differences in retirement age between sexes, we repeated the analysis in respondents younger than 60 years. The proportion of female respondents still ranged widely from 42·7% to 95·6% and did not significantly alter the outcome of the analysis (p < 0·001, Cramer’s v 0·29).

#### 3.2.2 Age

The mean age of respondents was 49.1 (SD 12.0) years (calculated from grouped data). The age distribution significantly differed between countries (country-specific mean age: lowest in Romania 43·4 years, and highest in Finland 55·3 years). However, the association was weak (Cramer’s v 0·18). The association between respondent age and GNI per capita was negligible.

#### 3.2.3 Speciality

On average, 93.0% of the respondents were endocrinologists, ranging between 64·2% in Finland and 100% in Belarus and Ireland (**Supplementary Figure 1**). The association between the proportion of endocrinologists and country was moderate (p<0·001, Cramer’s v 0·30). There was no association between the proportion of endocrinologists and GNI per capita or age.

#### 3.2.4 Place of work

The study included 2,181 (38·3%) physicians affiliated with university centres and 3,514 (61·7%) not affiliated with university centres (**Supplementary Figure 2**). The distribution of respondents affiliated with the university centres differed between countries, being lowest in Belarus (5·5%) and Ukraine (11·3%) and highest in Spain (75·7%) and Serbia (71·7%). The association was relatively strong (p<0·001, Cramer’s v 0·42). Neither sex nor GNI per capita was associated with the proportion of respondents affiliated with a university centre.

##### 3.2.4.1 Private practice

To analyse the differences between respondents who worked exclusively in private practice and those who did not, we excluded respondents who worked in both settings. The sample included 794 (16·2%) respondents working exclusively in private practice and 4,097 (83·8%) who did not (**Supplementary Figure 3**). The distribution of private practitioners differed between countries, and the association was relatively strong (p <0·001, Cramer’s v 0·40). The association between the proportion of private practitioners and sex was negligible (p=0·042, Cramer’s v = 0·03). There was a linear relationship between the proportion of private practitioners and age (Cochran-Armitage test for trend, p <0·001, two-sided). The age distribution differed between private practitioners and other respondents, but the association was weak (Fisher’s exact test, p <0·001, Cramer’s v 0·13). The proportion of private practitioners differed between geographic regions, and the association was moderate (p-value <0·001, Cramer’s v 0·23). Including respondents who worked in private and public practice did not significantly change the results of this analysis.

#### 3.2.5 Number of hypothyroid patients treated per year

A small minority of respondents (n=158, 2·8%) reported that they rarely treated hypothyroid patients, 787 (13·9%) treated 10-50 patients per year, 51-100 patients per year were treated by 1,206 (21·2%), and 62·1% (3,526) treated over 100 patients per year. This distribution differed between countries (**Supplementary Figure 4**). The lowest proportion of respondents who treated more than 100 patients per year was reported in Sweden, Finland and Denmark (15·5%, 22·0% and 27·2%, respectively) and the highest in Turkey, Greece and the Slovak Republic (92·2%, 86·7%, 83·7%, respectively). The association between country and number of treated patients per year was moderate (p <0·001, Cramer’s v 0·26).

### 3.3 Multivariate analyses

#### 3.3.1 Response rates, age, sex, survey language and GNI per capita

The mean age of respondents, the proportion of female respondents, survey language (local or English), GNI per capita, number of days the survey was accessible, and number of reminders sent were not associated with response rates (data not shown).

#### 3.3.2 Age, sex, country and geographical region

Logistic regression, where age was nested within the geographic region, showed a significant inverse relationship between age and proportion of female respondents in all regions (p <0·01, AUC 0·69, 95% CI: 0·68-0·71). After restricting age to less than 60 years, logistic regression, where age was nested within the geographic region, showed that the inverse relation between age and proportion of female respondents was lost in Northern Europe and Western Asia (logistic regression p <0·01, AUC 0·65, 95% CI: 0·64-0·67).

#### 3.3.3 Age, sex, private practice, number of hypothyroid patients treated per year, and GNI per capita

**Figure 3:**
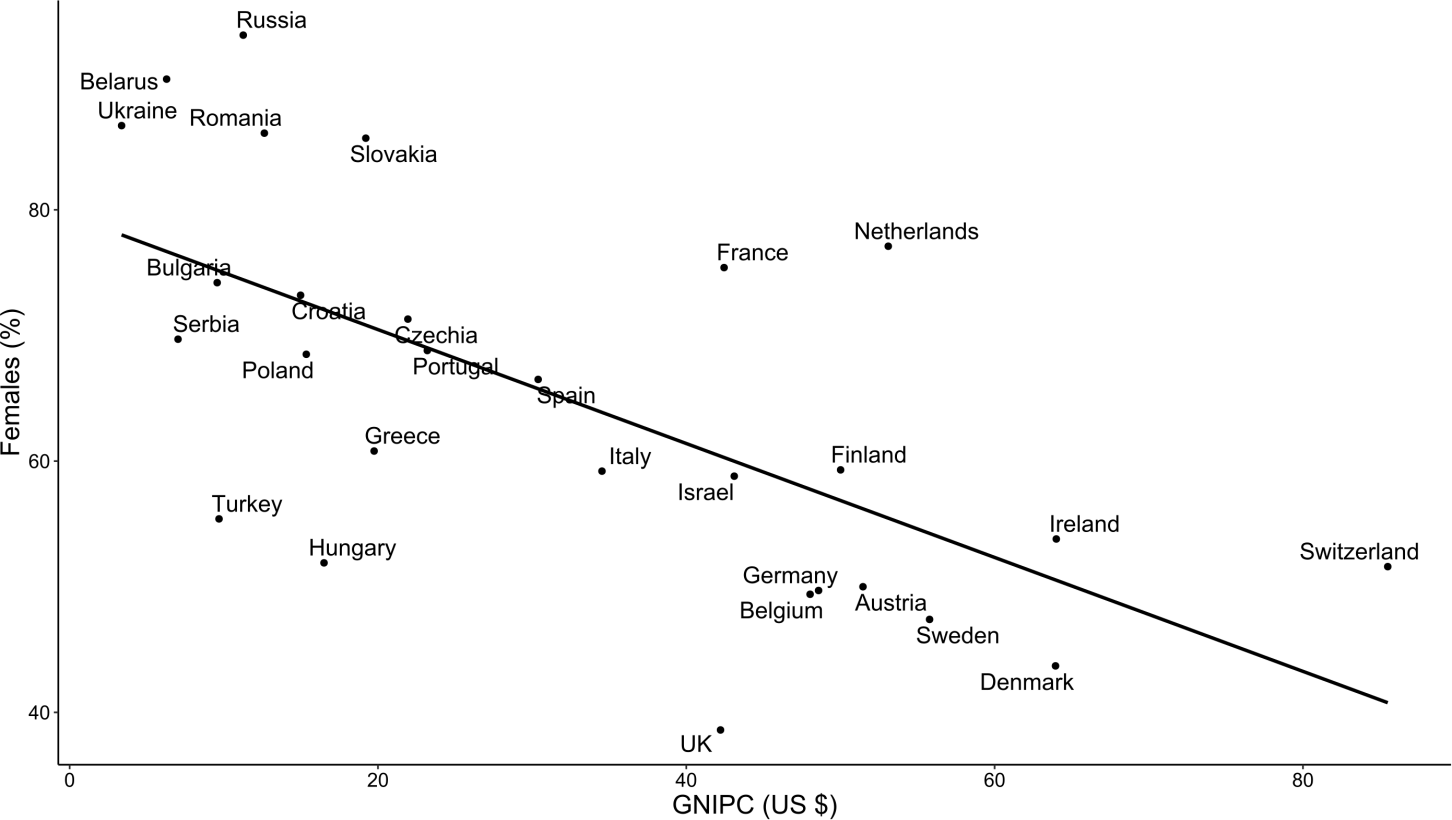
Relation between the proportion of female respondents and gross national income per capita (GNI per capita).

**Figure 4:**
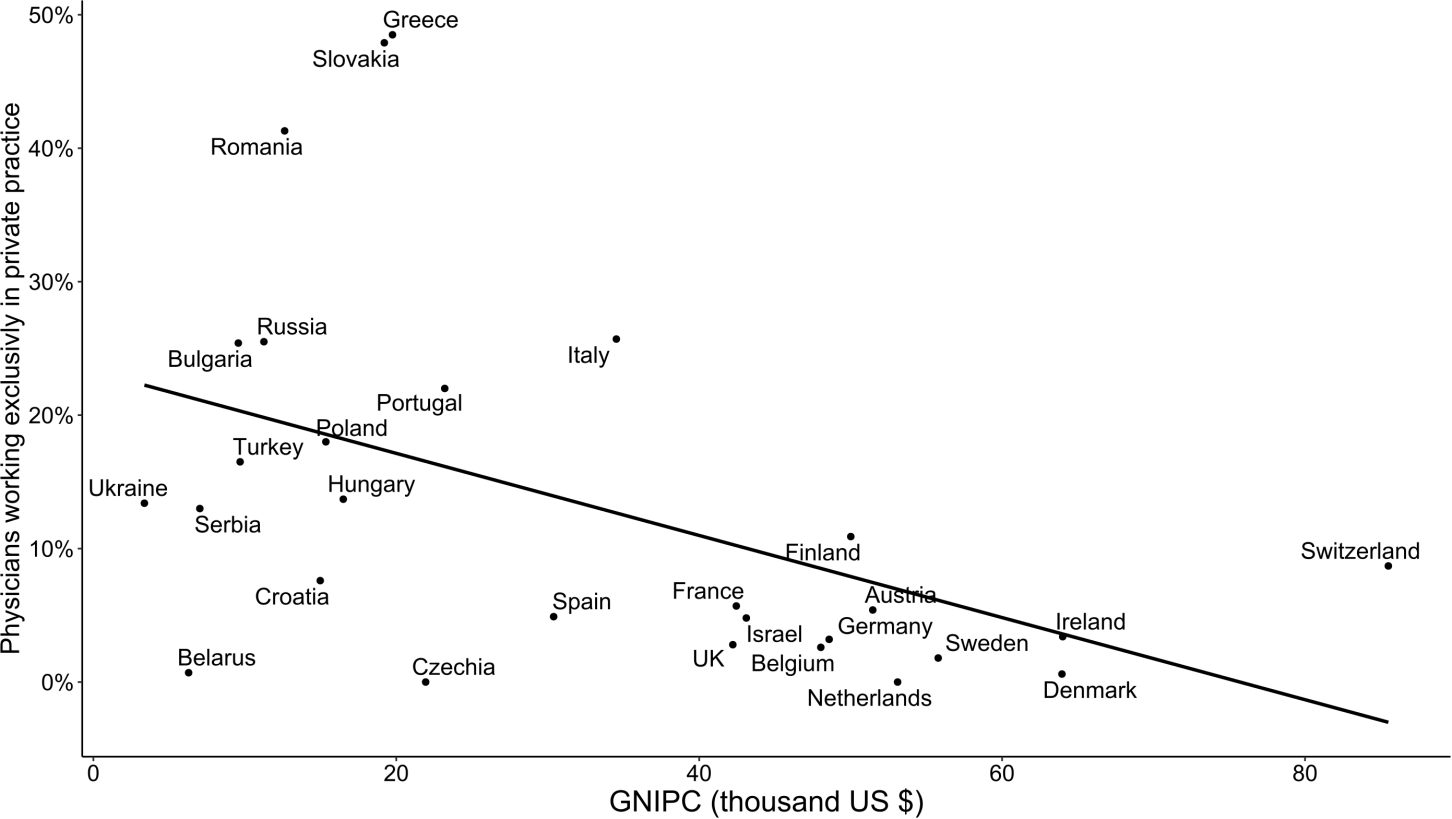
Relation between gross national income per capita (GNI per capita) and proportion of private practitioners.

A striking relationship was noted between respondent sex and national economic status (expressed as GNI per capita). GNI per capita was inversely associated with the proportion of female respondents (p <0·001 by linear regression, r^2^ = 0·42, **Figure 3**). Excluding respondents older than 60 years did not significantly alter this finding (p <0·001 by linear regression, r^2^ = 0·38). Decreasing GNI per capita correlated with an increasing proportion of private practitioners (p <0·011 by linear regression, r^2^ = 0·23, **Figure 4**). As GNI per capita increased, the proportion of respondents who treated more than 100 patients per year decreased (p <0·01 by linear regression, r^2^ = 0·36).

## 4 Discussion

Thyroid specialists play a key role in promoting public health by facilitating early diagnosis and treatment, educating patients on the importance of managing their condition, and collaborating with other healthcare providers to manage and control their patients’ hypothyroidism and other related health conditions. Thyroid specialists are also involved in research and development of guidelines. By addressing these public health aspects, thyroid specialists can improve the overall health and well-being of individuals and communities affected by hypothyroidism.

We surveyed thyroid specialists belonging to national endocrine and thyroid professional organisations of the most populous European countries to document the demographic and work-related characteristics of physicians who treat hypothyroid patients in different European countries and explore associations with geo-economic factors. The overall valid response rate of 33% is comparable to response rates achieved by European physician surveys related to topics focusing on benign thyroid disease since 2008 (median 38%, range 26-52%) (40–43). Comparing physicians’ characteristics (sex distribution by country) from Eurostat (39) with THESIS showed negligible differences (the largest Cramer’s v was 0·027). It has been suggested that the relationship between the survey response rate and non-response bias is small (44). Despite this, we assessed non-response bias using two different approaches (34, 45). One was to assess the influence of measured variables on response rate, and another was to compare the distribution of sample variables with the distribution of the same variables in the reference population. Neither of these approaches showed systematic bias, suggesting that the survey reflects a representative sample.

Moreover, the response rates recorded in THESIS are likely to be underestimated, as national endocrine and thyroid professional organisations include members who are not clinically active and are most likely not to have responded to the questionnaire. The participants are likely to represent European specialists who manage thyroid diseases. The response rate was not statistically dependent on the language used in the questionnaire (national or English language), age, sex of the respondent, allowed time for responding or GNI per capita, but did vary by country, being lowest in the Netherlands, Switzerland, and Austria (6·8-19%) and highest in Germany, Ireland, and Bulgaria (79·1-95·2%). Several reasons could explain the national differences in response rates. In many European countries, patients with hypothyroidism are treated preferably by general practitioners whereas thyroid patients with more complex pathologies are referred to an endocrinologist. Also, endocrine societies include members who do not treat thyroid patients (pathologists, basic scientists etc). Furthermore, thyroid specialists from some national professional organisations are more accustomed to completing surveys (for example the Italian Associazione Medici Endocrinologi) and achieve high response rates compared to others (46). Response rates may also have been negatively influenced by the availability of potential THESIS participants involved with the ongoing COVID-19 pandemic.

An important question is whether the data reported here are relevant to the bulk of care delivered to hypothyroid patients at large. Complementary surveys targeting primary care physicians will be required to address this. However, the participating thyroid specialists (which included national opinion leaders) are likely to exert a major influence on practices in primary care at the national level. Furthermore, it is anticipated that the population of hypothyroid patients managed by thyroid specialists represents those patients who experience difficulties in achieving therapeutic targets or have persistent symptoms and utilise services most. The latter subgroup of hypothyroid patients has been the subject of intense research in the past three decades. Being aware of variations in demographic and geo-economic parameters of health providers is of interest (5).

Most respondents were female (65%), although this varied widely across countries (39-93%), with the largest differences observed between Northern and Eastern Europe, the former showing the lower proportion of female endocrinologists. Restricting the analysis to respondents younger than 60 years (to overcome the potential bias due to the difference in retirement age for men and women) did not significantly change the results. The increase in the proportion of women physicians is a universal finding. Eurostat data show that more females are enrolled in medical schools than men, reaching 60% in 2021 (47). Low salaries may disincentivise male workers and instigate their relocation to other higher-paying employment or countries with a higher GNI per capita. Other possible explanations include increased educational opportunities for women, changing gender norms and attitudes, and supportive policies and initiatives to promote gender equality. However, the influence of the GNI per capita favours the former explanation.

Hypothyroid patients seem to be managed predominantly by young female thyroid specialists in Europe, as demonstrated by the significant inverse linear relationship between the proportion of female respondents and age. This tendency applies across Europe as the relationship was present and robust in all the participating regions and is supported by the Eurostat data. This accords with the global increase in the proportion of women in all medical fields (48). Our finding of a female preponderance, particularly in Eastern European countries, is consistent with a recent trend of rising female sex ratios among Eastern European University staff (49). It has been speculated that low salaries disincentive male workers and instigate their relocation to other higher-paying employments or countries with a higher GNI per capita (49). Our finding of an inverse relation between GNI per capita and the proportion of female respondents is consistent with this hypothesis.

THESIS has shown that the work environment of thyroid specialists differs between countries. Private practice, in particular, varied widely. Of note, GNI per capita was inversely associated with the proportion of private practitioners. This may be explained by the fact that European countries with high GNI per capita have more efficient and easily accessible national healthcare systems than less affluent countries, resulting in a less needed private health sector. Higher salaries for thyroid specialists employed by the public health system in wealthy countries also may reduce the drive to work privately. The linear relationship between age and proportion of respondents engaged in private practice probably reflects a switch by the more experienced (and hence older) physicians from public to private practice in their later careers. Finally, the number of treated patients per year differed between countries and was inversely associated with the GNI per capita. This, too, is likely related to wealthy countries having a well-established and efficient primary care tier that manages most patients with hypothyroidism. The strong association between thyroid specialist characteristics and GNI per capita raises the important question of whether hypothyroid patients are treated differently according to geo-economic factors, and merits further investigation.

Strengths of THESIS include a large number of responses from nearly all European nations with a population of more than 4 million, utilisation of reliable channels for dissemination of the questionnaire in the form of national endocrine and thyroid professional organisations, exploration of a topic that has never been studied in such detail before and the potential repercussions of thyroid specialist characteristics on the patient experience.

There are also limitations: the questionnaire was not validated; the questionnaire had to be translated into several languages; the time for responding was unstandardised and varied substantially between nations; some of the national data were published and available before the end of the study, thus potentially allowing some of the countries access to how others in other countries had responded; the response rates varied considerably between countries; the impact of the COVID-19 pandemic (50, 51). To mitigate bias, strict inclusion criteria were applied, and robust statistical analyses were employed. However, the shortcomings alluded to above may have influenced the results of THESIS.

Thyroid specialists in Europe are predominantly young female endocrinologists, most of whom work in the public sector. Striking inverse associations were shown between the female sex and GNI per capita and private practice and GNI per capita. The most significant contributor to national differences was GNI per capita, but organisational aspects of each healthcare system, historical influences, and socio-political factors might play a role. Demographic and geo-economic variations among specialist healthcare providers for patients with hypothyroidism in Europe are notable and may have a bearing on patient-reported outcomes.

## Data availability statement

The datasets presented in this article are not readily available because these are data from members of 28 European Endocrine Societies, currently forming the basis of a multitude of national as well as aggregate data publications. Requests to access the datasets should be directed to milos.zarkovic@med.bg.ac.rs and laszlo.hegedus@rsyd.dk.

## Author contributions

LH, EP, EN and PP conceptualized the study. MZ drafted the manuscript and performed the data analyses. MZ, LH EP, EN, RN, RA and PP participated in the interpretation of data, critically reviewed the manuscript, and approved the final draft. CAC, EA, MA, GA, TB, EB, MB, AB, MB, CB, MB, JC, JD, HD, VF, BF, EF, CF, DS, JG, TH, JJ, PK, MK, MK, MK, ML, IL, LL, VL, AM, MM, SM, CM, TM, TM, BB, DN, BP, TP, CP, ER, PR, MR, KR, AS, JF, DU, IV, WV, MV, YY, EY participated in data collection, critically reviewed the manuscript and approved the final draft. All authors contributed to the article and approved the submitted version.
