# Supplementary material for: Characteristics of specialists treating hypothyroid patients: the “THESIS” collaborative
Source: Front Endocrinol (Lausanne). 2023 Nov 7;14:1225202. doi: 10.3389/fendo.2023.1225202 (PMC10660282; doi:10.3389/fendo.2023.1225202)
Supplement: Supplementary file 1 [file DataSheet_1.docx]

Supplementary Material

# Supplemental Table 1. List of endorsing professional organisations

| Country | Endorsing professional organisation |
| --- | --- |
| Austria | Austrian Endocrine Society |
| Belarus | Belarus Public Med Assoc Endocrinol & Metabolism |
| Belgium | Belgian Thyroid Club |
| Bulgaria | Bulgarian Society of Endocrinology |
| Croatia | Croatian Society for Endocrinology |
| Denmark | Danish Endocrine Society |
| Czechia | Czech Endocrine Soc/Czech Med Association |
| Greece | Hellenic Endocrine Society |
| Finland | Endocrine Society of Finland |
| France | French Society of Endocrinology |
| Germany | Deutsche Gesellschafft für Endokrinologie |
| Hungary | Hungarian Society of Endocrinology and Metabolism |
| Ireland | Irish Endocrine Society |
| Israel | Israeli Endocrine Society |
| Italy | Associazione Medici Endocrinologi |
| Netherlands | Dutch Thyroid Network/Dutch Endocrine Soc |
| Serbia | Serbian Endocrine Society |
| Switzerland | Swiss Society of Endocrinology and Diabetes |
| Poland | Polish Endocrine Society |
| Portugal | Portuguese Soc Endocrinol Diab & Metabolism |
| Romania | Romanian Society of Endocrinology |
| Russia | Russian Association of Endocrinologists |
| Slovakia | Slovak Endocrine Society |
| Spain | Endocrine Society of Spain |
| Sweden | Swedish Endocrine Society |
| Turkey | Society of Endocrinol & Metabolism of Turkey |
| UK | Society for Endocrinology |
| Ukraine | Association of Endocrinologists of Ukraine |

# Supplementary Figures

## Supplemental figure 1. The percentage of respondents who declared themselves as an endocrinologist by country

## Supplemental figure 2. Percentage of respondents practising at the university centre by country

## Supplemental figure 3. Percentage of respondents practising only at the private practice by country

## Supplemental figure 4. Percentage of respondents treating more than 100 hypothyroid patients per year by country
